# *Mycobacterium setense* Infection in Humans

**DOI:** 10.3201/eid1408.080179

**Published:** 2008-08

**Authors:** Alexandre Toro, Toidi Adekambi, François Cheynet, Pierre-Edouard Fournier, Michel Drancourt

**Affiliations:** * Université de la Méditerranée, Marseille, France; † Centers for Disease Control and Prevention, Atlanta, Georgia, USA; ‡ Assistance Publique Hôpitaux de Marseille, Marseille, France

**Keywords:** Mycobacterium setense, rapidly growing mycobacteria, human infection, imipenem, letter

**To the Editor:** A 66-year-old man had a bone graft for treatment of an oroantral fistula in March 2007 in Marseille, France. The surgery consisted of a bilateral maxillary sinus filling with a parietal osseous graft to close the fistula (position 24–25). Painful edema of the hemiface and mild fever developed in the patient in July 2007. Computed tomography showed areas of hypodensity in the osseous graft in the left maxillary sinus consistent with osteolysis. Microscopic examination of a bone biopsy specimen after gram staining did not reveal any organisms but this specimen did grow *Enterobacter cloacae* and colonies of a gram-positive bacillus after a 2-day inoculation on 5% blood agar incubated at 37°C in an atmosphere of 5% CO_2_. Tentative identification of this catalase-positive, oxidase-negative gram-positive rod (isolate 74023791) by an API Coryne strip (API bioMérieux, La Balme-les Grottes, France) remained inconclusive. Isolate 74023791 exhibited acid fastness and was further identified by comparing its 16S rDNA (*1*) and heat shock protein 65 (hsp65) (*2*) sequences with those in the GenBank database and its RNA polymerase subunit B (*rpoB*) sequences (*3*) with those of *Mycobacterium* spp. in our local rpoB database.

Antimicrobial susceptibility testing using E-test (AB Biodisk, Bruz, France) indicated that isolate 74023791 was susceptible to ciprofloxacin with an MIC of 0.047 μg/mL and resistant to amoxicillin (MIC >256 μg/mL), ceftriaxone (MIC >256 μg/mL), erythromycin (MIC >256 μg/mL), clarithromycin (MIC >256 μg/mL), and rifampin (MIC >32 μg/mL). Disk testing and reference broth dilution method (*4*) showed that isolate 74023791 was susceptible to imipenem after a 3-day incubation. However, E-test showed heterogeneous resistance with colonies exhibiting an MIC >256 μg/mL. The same observations were made for *Mycobacterium setense* type strain CIP109395T (Collection de l’Institut Pasteur, Paris, France) (*5*). Daily treatment with 2 g imipenem and 1.5 g ciprofloxacin was prescribed for 1 month before the imipenem E-test and broth dilution results were available, and was followed by 1.5 g/day ciprofloxacin for 3 months, resulting in a favorable clinical and radiologic evaluation at 5-month follow-up.

Phylogenetic analyses indicated that isolate 74023791 belonged to the *M. fortuitum* group, along with *M. porcinum* and *M. conceptionense*, and was most closely related to M. *setense*, a recently described species of this group (*5*) (Figure). Isolate 74023791 shared 100% 16S rDNA (GenBank accession no. EU371507), 99.5% hsp65 (accession no. EU371508), and 99.0% *rpoB* (accession no. EU371509) sequence similarity with *M. setense* (accession nos. EF138818 and EU371504, EF138819 and EU371505, and EF414447 and EU371506, respectively). Because the 99% *rpoB* sequence similarity of the patient’s isolate was above the 97% rpoB sequence similarity cut-off value used to identify rapidly growing mycobacteria (*3*), isolate 74023791 was therefore identified as *M. setense*.

**Figure:**
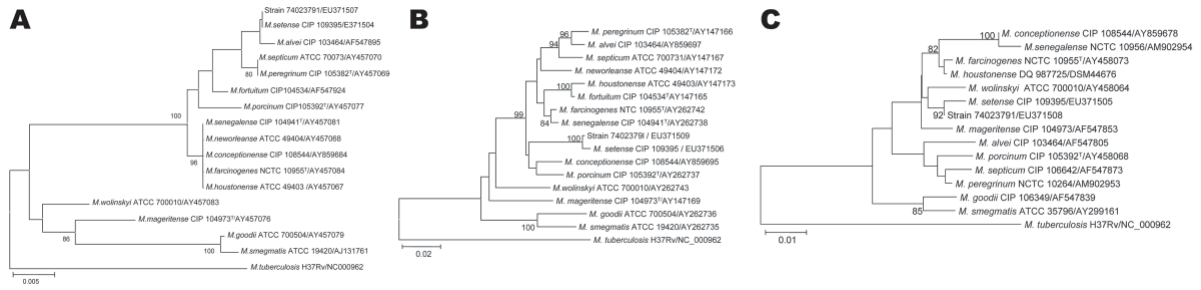
Phylogenetic position of isolate 74023791 and 16 rapidly growing *Mycobacterium* species based on A) 16S rDNA, B) partial RNA polymerase subunit B, and C) partial heat shock protein 65 sequences analyzed by using the neighbor-joining method and Kimura's 2-parameter distance correction model. The support of each branch, as determined from 1,000 bootstrap samples, is indicated by the value at each node when >80% (as a percentage). *M. tuberculosis* was used as the outgroup species. Scale bars represent differences in nucleotide sequences.

*M. setense*, in association with an *E. cloacae* strain susceptible to antimicrobial drug therapy, was an agent of infection in our patient. It is noteworthy that *M. setense* and the closely-related species *M. conceptionense* were isolated from patients with posttraumatic osteitis (*5*,*6*); *M. porcinum* was isolated from 7/46 cases of osteomyelitis and additional cases of postsurgical infection, respectively (*7*); *M. fortuitum* osteomyelitis has also been reported (*8*). These data emphasize the role of *M. fortuitum* group organisms in posttraumatic and postsurgical osteitis.

In a later interview, the patient disclosed that he rinsed his mouth with well water during the weeks after receiving the bone graft. We initially suspected that the water was the source of *M. setense*, as previously suspected for *M. conceptionense* (*6*) and reported for *M.* *porcinum* (*7*). However, neither *M. setense* nor *M. setense* DNA were detected in the well water in October 2007.

Initially, *M. setense* was reported to be susceptible to imipenem by the disk diffusion method, which is not the reference method (*5*). In this report, the disk and reference broth dilution methods showed that both clinical and reference *M. setense* strains were initially susceptible to imipenem but the E-test disclosed that both strains exhibited heterogeneous resistance to imipenem; colonies exhibited an MIC >256 μg/mL. This result was unexpected because the E-test showed that related species *M. fortuitum* CIP104534T, *M. conceptionense,* CIP 108544T and *M. porcinum* CIP105392T were susceptible to imipenem (*6*). Likewise, the modified broth dilution method showed that the MIC for imipenem was <4 μg/ml for *M. fortuitum* (*9*). However, when a broth dilution method was used, the MIC for imipenem ranged from 0.5 μg/mL to 8 μg/mL in 42 *M. porcinum* strains (*7*). Together, these data challenge the susceptibility to imipenem in organisms of the *M. fortuitum* group.

*M. setense* is an emerging organism of the *M. fortuitum* group that must be added to the growing list of rapidly growing mycobacteria isolated from humans. The initial gram-positive rod appearance of *M. setense* may delay its accurate identification. Determination of antimicrobial drug susceptibility needs to be conducted by the reference broth dilution method. Further reports are warranted to characterize the role of *M. setense* in infection.
